# GenAI exceeds clinical experts in predicting acute kidney injury following paediatric cardiopulmonary bypass

**DOI:** 10.1038/s41598-025-04651-8

**Published:** 2025-07-01

**Authors:** Mansour Sharabiani, Alireza Mahani, Alex Bottle, Yadav Srinivasan, Richard Issitt, Serban Stoica

**Affiliations:** 1 School of Public Health, Imperial College London, London, UK; 2 New York Stock Exchange, New York, United States; 3 Great Ormond Street Hospital, London, UK; 4 Bristol Royal Hospital for Children, Bristol, UK

**Keywords:** Generative artificial intelligence, Text embedding, Electronic health records, Cardiopulmonary bypass, Acute kidney injury, Spherical k-means, Acute kidney injury, Statistics

## Abstract

The emergence of large language models (LLMs) opens new horizons to leverage, often unused, information in clinical text. Our study aims to capitalise on this new potential. Specifically, we examine the utility of text embeddings generated by LLMs in predicting postoperative acute kidney injury (AKI) in paediatric cardiopulmonary bypass (CPB) patients using electronic health record (EHR) text, and propose methods for explaining their output. AKI could be a serious complication in paediatric CPB and its accurate prediction can significantly improve patient outcomes by enabling timely interventions. We evaluate various text embedding algorithms such as Doc2Vec, top-performing sentence transformers on Hugging Face, and commercial LLMs from Google and OpenAI. We benchmark the cross-validated performance of these ‘AI models’ against a ‘baseline model’ as well as an established clinically-defined ‘expert model’. The baseline model includes structured features, i.e., patient gender, age, height, body mass index and length of operation. The majority of AI models surpass, not only the baseline model, but also the expert model. An ensemble of AI and clinical-expert models improves discriminative performance by 23% compared to the baseline model. Consistency of patient clusters formed from AI-generated embeddings with clinical-expert clusters—measured via the adjusted rand index and adjusted mutual information metrics—illustrates the medical validity of LLM embeddings. We create a reverse mapping from the numeric embedding space to the natural-language domain via the embedding-based clusters, generating medical labels for the clusters in the process. We also use text-generating LLMs to summarise the differences between AI and expert clusters. Such ‘explainability’ outputs can increase medical practitioners’ trust in the AI applications, and help generate new hypotheses, e.g., by studying the association of cluster memberships and outcomes of interest.

## Introduction

While predictive models have traditionally relied on structured features—including those defined by domain experts—advances in large language models (LLMs) offers new opportunities to leverage unstructured data—e.g., clinical notes in electronic health records (EHRs)—as a supplement to structured features for predictive modelling in healthcare^1,2^. This study aims to assess the utility of text embeddings generated by LLMs in predicting acute kidney injury (AKI) in paediatric CPB patients, and to explore methods for explaining their output and improving their predictive power. Postoperative AKI is a critical complication in paediatric patients undergoing cardiopulmonary bypass (CPB) and thus accurate prediction of AKI can significantly improve patient outcomes by enabling timely interventions.

**Figure 1:**
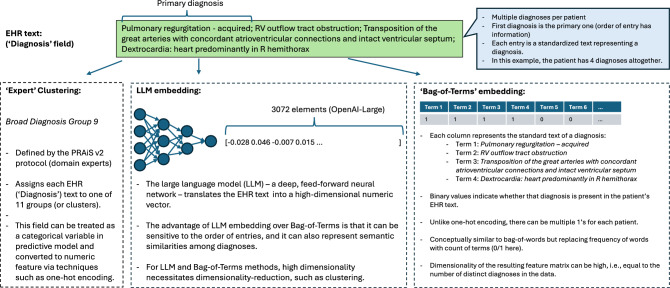
Illustration of three approaches for embedding EHR text: expert mappings according to PRAiS v2 (left), embedding by LLMs (middle), 'Bag-of-Terms’ embedding (right). The green box shows an example text for the ‘Diagnosis’ field, consisting of a a primary diagnosis, followed by several more, separated by ';'.

Text embedding involves converting text – whether a single word, or a sequence of words forming a sentence, paragraph, or an entire article – into a numeric vector. Embedding techniques range from simple—e.g., Bag-of-Words (BoW) or term frequency-inverse document frequency (TF-IDF)—to advanced (e.g., transformer-based LLMs). Modern text embedding models often produce high-dimensional vectors that are designed to preserve contextualised semantics of the input (natural language). Embedding a text string produces a vector (Fig. 1), and thus embedding a text column results in a numeric matrix. Each column of this matrix can be treated as a feature in a predictive model.

### Related work

Machine learning (ML) and artificial intelligence (AI) have been extensively used for predicting acute kidney injury (AKI), leveraging various data modalities and model architectures. For instance^3^, developed a Paediatric Early AKI Risk Score based on structured electronic health record (EHR) data in a paediatric ICU setting. In contrast^4,5^ utilised unstructured clinical notes from the MIMIC-III database, applying text embedding methods such as TF-IDF and BoW to generate predictive features. The latter also integrated structured data such as laboratory results.

Reference^6^ predicted AKI in emergency departments using only structured EHR data, while^7^ fine-tuned a Bidirectional Encoder Representations from Transformers (BERT) model for analysing clinical notes, demonstrating improved prediction performance. Additionally^8^, focused on cardiac surgery-associated AKI, comparing multiple ML models trained on perioperative structured data and highlighting the role of intraoperative variables. Finally^9^, summarised ML models for AKI prediction in critical care, emphasizing the challenges of model integration into clinical practice.

In a recent study^10^, applied large language models (LLMs) to embed clinical notes for predicting multiple postoperative outcomes, including AKI, and highlighted the impact of fine-tuning strategies on prediction accuracy.

Applications of text embeddings in healthcare extend beyond AKI prediction. For example^11^, modelled patient trajectories and disease progression using embeddings derived from clinical text. Reference^12^ surveyed word embeddings for clinical text, discussing their use in tasks like predicting unplanned readmission and ICD code assignment. Similarly^13^, compared classical ML and deep learning models for morbidity prediction, employing embeddings such as word2vec and GloVe.

In sepsis prediction^14^, conducted a systematic review and found that combining text and structured data improved early detection compared to structured data alone. Moreover^15^, proposed a generative AI-based named entity recognition (NER) system for extracting entities from unstructured medical narratives, showcasing the broader potential of LLMs in medical text analysis.

### Our contribution

While previous work has demonstrated the utility of text embeddings in healthcare in general, and in predicting postoperative outcomes such as AKI in particular, our focus on the following themes in this research makes for a novel and practical contribution to the field:

- **Use of baseline structured features:** Beyond unstructured text, we integrate patient demographics and operative details (e.g., gender, age, height, weight, operation time) to reflect real-world clinical scenarios where structured variables are routinely available.
- **Emphasis on explainability:** Our framework combines soft cluster memberships in predictive models with hard cluster labels for interpretative summaries, both derived from the *same* LLM-based embeddings. This dual use enhances trust by linking model predictions to interpretable patterns.
- **Structured medical terms as benchmarks:** Using a pediatric cardiopulmonary bypass (CPB) dataset, we benchmark embeddings against expert-defined clusters (Broad Diagnosis Grouping and Transformed Specific Procedure) and a straightforward ‘bag-of-terms’ representation (see Methods), demonstrating the added value of advanced embeddings and providing an objective assessment of the medical informativeness of LLM embeddings.
- **Evaluation of state-of-the-art LLMs:** We benchmark top-performing open-source and commercial LLMs—including models from Google and OpenAI—highlighting recent advancements in text-embedding technologies.

In summary, our study provides a comprehensive framework that integrates unstructured texts via LLM embedding and clustering with standard patient attributes, showing that modern LLM embeddings can surpass expert-defined features—or combine with them—in achieving superior prediction of AKI.

For a list of technical terms and their definition, please see Glossary.

## Methods

### Data collection and ethical approval

This work is a retrospective analysis of consecutive patients aged younger than 18 years (maximum age treated at the institution) undergoing 963 CPB surgeries at Great Ormond Street Hospital between 2019 and 2021. Clinical data were collated in a research platform within the hospital’s governance structure and de-identified before analysis. Institutional approval was obtained from the Great Ormond Street Hospital for conducting this study (audit number 3045). Due to the retrospective nature of the study, the NHS Health Research Authority London–Bloomsbury Research Ethics Committee (REC reference: 17/LO/0008) waived the need for obtaining informed consent. All methods were performed in accordance with the relevant guidelines and regulations. Further details on data collection can be found in^16^.

We acknowledge the imbalance in our dataset, particularly in the binary AKI outcome (KDIGO-Binary), where 26% of patients experienced severe AKI. To assess if this imbalance impacted the generalizability of our model, we employed robust validation methods, specifically repeated cross-validation with 250 repetitions and 10 folds each. This rigorous validation approach ensures that model performance metrics are reliable and indicative of genuine predictive capability despite class imbalance. Additionally, the use of metrics such as area under receiver operating curve (ROC-AUC), which is robust to class imbalance, further ensures that our model’s reported predictive performance generalizes well.

### Data preparation

A set of ‘baseline’ patient attributes were included in this analysis: gender, age, height and weight, as well as the length of operation. Note that age, height and weight were all recorded as of the start of operation. Weight and height were transformed to BMI and ‘residual’ height. The former followed the standard definition, while the latter was defined as the percent deviation of height from the predictions of a LOESS (locally estimated scatterplot smoothing) model^17^ that regressed height on age and gender.

The EHR text data included patient diagnoses (diagnosis), and operations performed (operation). Each text field value is an ordered set of standardised medical codes, with the first entry representing the primary code. Expert-defined clusters for diagnosis (called, ‘broad diagnosis grouping’ or BDG) and operation (called, ‘transformed specific procedure’ or TSP) were also obtained using the Partial Risk Adjustment in Surgery (PRAiS2) protocol^18^. PRAiS2 is a model developed in the UK to predict 30-day mortality risk after paediatric heart surgery. The BDG and TSP groupings are defined and used in the PRAiS2 risk model as categorical variables. Figure 1 provides an example for the operation field.

For outcome, we focus on postoperative AKI. In particular, we consider the following five outcomes:

- *Creatinine ratio*: Ratio of postoperative to baseline serum creatinine.
- *Urine output (0.5)*: Length of time (in hours) within the 48 hours after CPB when patient’s urine output per hour is below 0.5 ml per kilogram of body mass.
- *Urine output (0.3)*: Same as above, but comparing urine output to a 0.3 ml per kilogram of body mass (per hour) threshold.
- *KDIGO-Ordinal*: An integer, ordinal score from 0 to 3, defined from using a combination of the above three numbers, per KDIGO protocol^19^. For instance, a value of 1 is assigned to a patient if their postoperative-to-preoperative creatinine ratio is between 1.5-1.9 or the length of time with a urine output below 0.5 ml/kg/h is more than 6 hours but less than 12 hours during the 48-hour period after the operation.
- *KDIGO-Binary*: A binary indicator of severe AKI. It is 1 if KDIGO-Ordinal is 2 or 3, and 0 if KDIGO-Ordinal is 0 or 1. This choice was driven by the observation that most cases of KDIGO-Ordinal=1 are quite mild and achieve full recovery, and predicting the severe cases is a more clinically relevant task.

For predictive tests, we focused on ‘KDIGO-Binary’ as outcome, while for explainability analyses, we considered all five outcomes. After removing operations with missing values, we are left with 780 data points for predictive models. Table 1 provides a summary of the data.

**Table 1:** Summary of dataset used in the predictive models presented in this paper. Numbers in parentheses indicate percentages of total data. Numbers in brackets represent the interquartile range. Note that for 30-day mortality calculation, sample size is slightly smaller (757).

Variable	Value
Number of operations	780
Number of unique patients	736
KDIGO-Ordinal	
0	315 (40)
1	266 (34)
2	121 (16)
3	78 (10)
KDIGO-Binary	
0	581 (74)
1	199 (26)
Gender	
Female	352 (45)
Male	428 (55)
Median age (months) at operation	8.3 [3.1–43.5]
Range	0.1–188.3
Median BMI at operation	15.3 [13.6–16.7]
Range	8.3–32.9
Mean/std of height residual	-0.001/0.07
Median operation time (minutes)	86 [57–122]
Range	10–471
30-day mortality (757 operations)	
Observed	2 (0.26)
Predicted (PRAiS2)	11 (1.5)

### Software and compute environment

The initial data preparation steps were performed in R 4.4.0^20^. All remaining steps were performed in Python 3.11.7. All Python and R scripts were executed on a 64-bit Windows 11 PC running on Intel(R) Core(TM) 7 150U 1.80 GHz, with 16 GB of installed RAM.

### Embedding algorithms

Text entries are transformed into numeric vectors using various encoding/embedding algorithms:

1. *Bag-of-Terms (BoT)*: Each medical term gets a binary indicator, and each text field entry gets 1’s for all medical terms present in the string, and 0’s for others (Fig. 1). The output dimension is thus dictated by the number of distinct medical terms used for each field, which is 282 and 179 for diagnosis and operation, respectively. BoT is a generalisation of one-hot encoding, where multiple levels of a categorical variable are not mutually exclusive. It is also similar to the standard BoW approach, where each medical term plays the role of a word. (However, unlike BOW, in BoT the entries are binary, i.e., the same code cannot appear more than once in a text field.) Note that using the BoT method is possible in our problem since—as illustrated earlier—the text fields are not free-format, but rather a collection of standardised entries. BoT serves as a reference, against which more sophisticated embedding algorithms (discussed below) are benchmarked.
2. *Doc2Vec*^21^: We used the implementation by the gensim Python package. Both distributed bag-of-words (DBOW) and distributed memory (DM) versions were tested, using values of 10, 100, 1000 for number of training epochs, and values of 40, 400 and 4000 for output dimension (i.e., length of embedding vector).
3. *Open-Source LLMs*: We used the publicly-available, top-ranking models from the Massive Text Embedding Benchmark (MTEB) leaderboard for embedding models^22^, plus PubMedBERT (which is a medically-specialised embedding model), MiniLM-L6 (which is a small model built on a distilled, pre-trained model) and ClinicalBERT. The models were downloaded from the Hugging Face repository using the sentence-transformers Python package. With the exception of the last model, all others have been fine-tuned for embedding tasks. For ClinicalBERT, we added mean pooling to the last hidden layer to convert token embeddings to sentence embeddings. While it is technically more appropriate to refer to the sentence-transformer model built on ClinicalBERT as ClinicalBERT+mean pooling, hereafter we will refer to this model as simply ClinicalBERT for brevity. Supplementary Material E contains the full names and revision numbers of Hugging Face models used in the paper.
4. *Commercial LLMs*: We used OpenAI and Google embedding LLMs via their web APIs, accessed through Python packages openai and vertexai, respectively. OpenAI offers three embedding models,
  text-embedding-3-large, text-embedding-3-small and
  text-embedding-ada-002, with output dimensions of 3072, 1536 and 1536, respectively. Hereafter, and for brevity, we will refer to them as OpenAI-large, OpenAI-small and OpenAI-ada, respectively. For Google, we used two models, text-embedding-004 (or Google-004 for short), and textembedding-gecko@003 (Google-gecko for short), both with an output dimension of 768. To use Google’s embedding models, we must also specify a ‘task’ parameter, with options being RETRIEVAL_QUERY, RETRIEVAL_DOCUMENT, SEMANTIC_SIMILARITY, CLASSIFICATION, CLUSTERING, QUESTION_ANSWERING and FACT_VERIFICATION. (The last three tasks are only applicable to the Google-004 model.)

### Fine-tuning

We defined the following fine-tuning task from the diagnosis and operation columns for the embedding models: We required the embeddings of the diagnosis and operation fields for each patient be closer to one another, compared to when we mix-and-matched the two field from two different patients. The underlying assumption is that the diagnosis field is the driver or cause of the operations performed on the patient.

These ‘diagnosis-operation’ pairs (963) were used to fine-tune three LLMs: ClinicalBERT, PubMedBERT and MiniLM-L6. The loss function used was MultipleNegativesRankingLoss in the sentence-transformers library. In a nutshell, this loss function forces the embedding vectors for matching diagnosis/operation pairs to be more similar (measured by their cosine distance) than non-matching pairs formed by random shuffling of operations and diagnoses in the same batch. We used the following parameters for fine-tuning the LLM: maximum number of epochs (20), learning rate (1e-6), warmup ratio (0.1), fraction of data used for early stopping (0.25), early-stopping patience (3 epochs), and early-stopping threshold (0.0).

### Spherical K-means clustering

We use ‘spherical’ k-means^23^, which we implemented in Python, for clustering patients according to their text embedding vectors. This is a variant of standard k-means, where the metric used to measure the distance between two vectors $\textbf{x}$ and $\textbf{y}$ is changed from L2 norm (i.e., $(\textbf{x} - \textbf{y})^T (\textbf{x} - \textbf{y})$) to ‘cosine distance’, $D_C(\textbf{x}, \textbf{y})$:

1 $$\begin{aligned} D_C(\textbf{x}, \textbf{y}) = 1 - \frac{\mathbf {x^T} \textbf{y}}{(\mathbf {x^T} \textbf{x})^{1/2} \, (\mathbf {y^T} \textbf{y})^{1/2}} \end{aligned}$$

This metric is invariant with respect to the length of the two vectors, focusing only on their angles and thus making it a suitable choice for text embedding vectors that are L2-normalised, i.e., $\mathbf {x^T} \textbf{x} = 1$. (Note that while L2 and cosine distances would produce identical assignments of points to clusters, they would differ in the centroid update step of the k-means clustering algorithm.) One exception is the BoT method, which does not produce L2-normalised vectors (in fact the number of medical codes present in the text field for a given patient—which is equal to the L1 norm of the BoT vector—has potentially relevant information), and hence we applied standard k-means clustering to BoT embeddings, rather than spherical k-means.

### Cluster similarity metrics

We use two symmetric metrics to quantify the degree of similarity between two clustering algorithms: 1) adjusted rand index (ARI)^24^, and 2) adjusted mutual information (AMI)^25^. In both cases, the adjustment subtracts a baseline value to account for the possibility of ARI/AMI values occurring by chance, and also divides by the possible range of the value to achieve a normalised score that has an upper bound of 1, and produces values close to 0 for two clusters with no concordance. Calculations were done using the metrics module of the scikit-learn Python package.

### Predictive contribution of embeddings

To quantify the contribution of text embeddings towards predicting AKI, we perform spherical k-means clustering—as discussed earlier - on the embedding vectors produced by the LLMs and other embedding algorithms listed in Embedding Algorithms. We then proceed to include the cluster ‘soft’ membership data as features—alongside the baseline features mentioned in Data Preparation—in a binary classification model. (We used logistic regression and ML models including random forest and neural networks.) The soft membership feature vector is simply a vector of distances between a given feature vector and the cluster centres, while hard membership is the cluster assignment of that observation, which translates into a binary vector after one-hot encoding. (It must be noted that, while here we report on results of using the soft cluster memberships as features, we also experimented with using the hard memberships, and the performance—measured by ROC-AUC—was worse.) We compare the ROC-AUC of models trained on cluster features based on different embedding models (the ‘AI models’) against each other, as well as against a model that uses expert clusters instead of embedding-based clusters (the ‘expert model’). In both types of models, we separately train two models, one using the diagnosis clusters alongside the baseline variables, and one using the operation clusters added to baseline variables. The predicted probabilities of these two models are averaged to form the final prediction.

### Hyperparameters

As mentioned in Embedding Algorithms, two of the embedding algorithms that we used have hyperparameters: Doc2Vec (number of training epochs, output dimension) and Google (embedding task). In both cases, we calculate the average ROC-AUC across all CV folds for each hyperparameter value or combination (and within each sub-model, such as the DBOW and DM versions of Doc2Vec), and choose the hyperparameter value that produced the highest ROC-AUC.

There are two other important hyperparameters in our experiments. First is the number of clusters in (spherical) k-means. For cluster consistency expeirments, we chose this to be equal the number of expert clusters (which is 11 for diagnosis and 15 for operation). This removes the confounding effect of the number of clusters from calculations of the ARI/AMI consistency metrics. In the predictive models, we used a round value of 10 for both text fields. The second hyperparameter is the number of dimensions of the embedding vector to include in downstream steps. For instance, one can choose the first 100 elements of a 768-dimensional embedding vector, or apply dimensionality-reduction techniques such as Principal Components Analysis. Here, we chose to always feed the entire embedding vector to the downstream clustering algorithms. Tuning these two hyperparameters and studying their effect on consistency of clusters with experts and/or predictive performance of models will be a topic of future research.

### Ensemble models

We used a simple, weighted-average ensemble approach, applied to the predicted probability of severe AKI produced by each constituent of the ensemble model. In the ‘AI Ensemble’, two models were included with 2:1 weights: OpenAI’s text-embedding-3-large (weight of 2) and Google’s textembedding-gecko@003 using the task CLASSIFICATION (weight of 1). In the ‘AI + Expert’ ensemble, the Expert model was added to the AI ensemble, with a weight equal to 1/10th of OpenAI’s weight. These weights were chosen based on limited trial-and-error. Applying more sophisticated ensemble techniques is another topic for future research.

### Explainability

Given the current architecture of LLMs and the unidirectional nature of their embedding vector generation, it is mathematically impossible to reverse-map an embedding vector back to its unique source text. This limitation prevents the direct transformation of cluster centroids into meaningful labels, such as core medical summaries for the clusters. To address this challenge, we developed an innovative two-step approach that involves a) grouping the original text based on cluster memberships, and then, b) utilising AI to generate meaningful and distinct medical labels for these text groups, effectively capturing the essence of each cluster in medical terms. In other words, our method mimics a reverse transformation, bridging the numeric domain of embedding vectors to the natural language domain.

More specifically, we asked the same OpenAI model as well as Google’s gemini-1.5-pro model to provide descriptive and distinct labels for clusters formed from a concatenation of diagnosis and operation embeddings. The prompt consists of two sections: 1) the preamble or instructions to the LLM, and 2) the body, containing data in numbered clusters that must be described by the LLM. Below is the instructions paragraph, corresponding to the case where clusters have been created using both diagnosis and operation fields:

**Table:** 

Prompt Instructions	
The following is a list of 963 pediatric patients undergoing cardiopulmonary bypass. Each row contains one or more surgical procedures, separated by ’;’. These are followed by one or more diagnoses, also separated by ’;’. Patients have been grouped into 10 groups, according to their diagnoses and procedures. Please suggest group labels that are representative of their members, and also distinct from each other:	

The body consists of group (or cluster) number, followed by observations in that group. For each observation, the value of the relevant text field(s) is are printed. In cases where we combine multiple text fields, we preface each text field with the name ofthe field. Below is an excerpt to illustrate the point. (*Italics* and **boldface** are added for highlighting in the manuscript, and prompts submitted to LLMs are plain text.)

**Table:** 

Prompt Body	
**Group 1:**	
*Operations*: aortic root replacement: valve sparing technique || *Diagnoses*: aortic regurgitation; congenital anomaly of aortic valve; doubly committed juxta-arterial ventricular septal defect (vsd) with anteriorly malaligned fibrous outlet septum and perimembranous extension	
*Operations*: scimitar syndrome (partially anomalous pulmonary venous connection) repair || *Diagnoses*: partial anomalous pulmonary venous connection of scimitar type	
*Operations*: aortic valvar replacement using mechanical prosthesis; left ventricular outflow tract obstruction relief || *Diagnoses*: aortic regurgitation; lv outflow tract obstruction; aortic valvar stenosis—congenital; discordant va connections (tga); superior caval vein (svc) abnormality	
*Operations*: mitral valvar replacement; mitral valvar annuloplasty || *Diagnoses*: mitral regurgitation	
*Operations*: mitral valvar procedure || *Diagnoses*: mitral regurgitation; mitral valvar abnormality; mitral valvar prolapse	
...	
...	
=======	
**Group 2:**	
*Operations*: vsd closure; patent arterial duct (pda) closure: surgical || *Diagnoses*: perimembranous central ventricular septal defect (vsd); patent arterial duct (pda)	
...	
...	

In this task, we used the ‘structured output’ features of OpenAI and Google to ensure that the LLM response was formatted in a predictable structure. This allows for automated creation of downstream tables and figures. Details can be found in Supplementary Material B. This explainability task is completed by computing the rank correlation—using Kendall’s tau—of the five AKI-related outcome metrics defined in “Data preparation” vs. membership in each of the embedding-based clusters.

In a second explainability application, we asked OpenAI’s gpt-4o model to summarise the differences between the AI-based clusters and the corresponding expert clusters (BDG and TSP labels). A detailed description of the prompt is provided in Supplementary Material A.

Note that both of the above explainability tasks involve long prompts (e.g. > 50,000 tokens) that would exceed the maximum context length of all but the latest generations of OpenAI and Google models.

### Process overview

**Figure 2:**
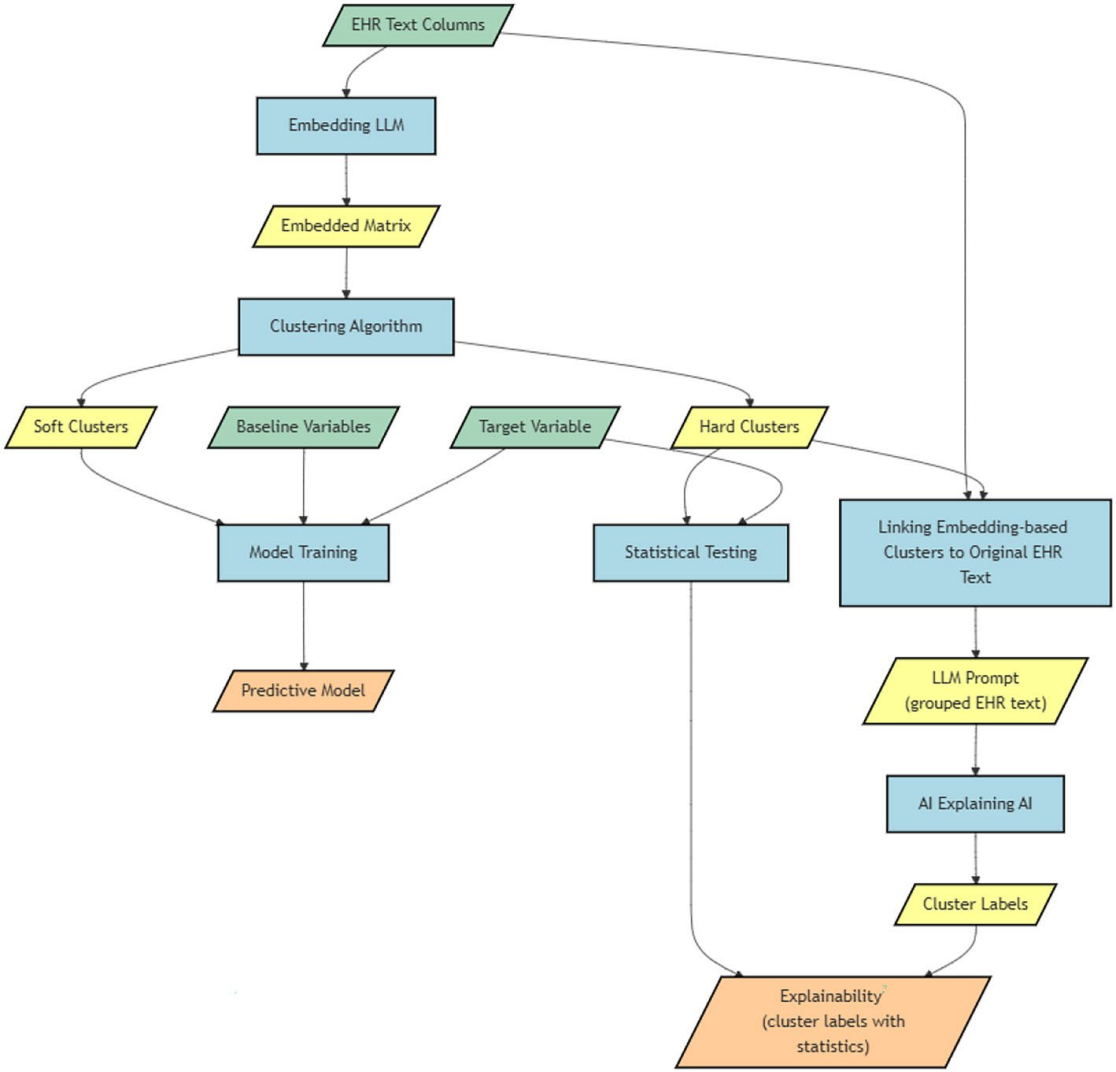
Overview of the methodology proposed in this paper for utilising EHR text alongside structured features. It shows how LLMs can be used for feature extraction from EHR text and for enabling explainability of predictive models using those features.

Figure 2 contains a visual summary of the methodology proposed in this paper for using text data—via LLM embeddings—alongside structured features in predictive models, and for explaining the features extracted by the LLMs from text. While the methodology has been developed in the context of healthcare analytics and EHR text, it can be applied to—and tested in—other domains.

### Benchmarking

To quantify the consistency of AI and expert clusters, we took the average ARI/AMI scores from 10 runs of spherical k-means. Within each run, we used 10 random initialisations of cluster centroids, where the cluster with the smallest total within-cluster distance is selected. Number of clusters was pre-selected to match the number of expert clusters (11 for diagnosis and 15 for operation).

In predictive experiments, we generate a single set of clusters from embedding vectors, using 100 random initialisations of spherical k-means with number of clusters set to 10. We used 250-times repeated, 10-fold cross-validation to compare the out-of-sample performance of different models. To maximise comparability of results, we fixed the random seed when calling the RepeatedKFold function of scikit-learn. For each run, therefore, we obtained 2500 sets of metrics, which we use to perform statistical analyses such as paired t-test. We report ROC-AUC, accuracy, sensitivity, specificity, precision and F1. For threshold-sensitive metrics (all except ROC-AUC), we report the average of the metric for threshold values from 0.1 to 0.9, in steps of 0.1.

To quantify the stability of AI clusters, we applied spherical k-means to concatenated embeddings of diagnosis and operation fields, produced by the OpenAI-large model. This was repeated 10 times, each time using 100 random starts and 10 clusters. We then calculated average ARI/AMI scores between all 45 (10$\times$9/2) unique pairs of clustering runs. The clusters were mapped across runs using a greedy algorithm for maximising member overlap. See Supplementary Material C for the source code and explanation of the cluster mapping algorithm.

Next, for each of the 10 clustering runs, we used a similar process to that outlined in the Explainability to solicit cluster labels from OpenAI and Google models. We split the 10 clustering runs evenly between OpenAI and Google. Labels were aligned across runs using the mapping generated per above.

## Results

The median age of patient population was 8.3 months, ranging from 0.1 to 188.3 months, with balanced gender representation (45% female). Table 1 presents further demographic details including BMI distribution, severity of postoperative AKI according to KDIGO scores, and details about surgical procedures. Observed mortality rate is below the level predicted for this patient population, according to the PRAiS2 protocol.

### Consistency of AI and expert clusters

Table 2 shows the ARI and AMI scores between AI clusters formed from operation and diagnosis text fields and their expert counterparts. The positive values of these metrics—which remove a baseline effect due to chance—indicate a statistically significant, although imperfect, consistency between the two approaches in extracting information from the underlying text fields.

**Table 2:** Consistency between ‘expert’ clusters (rows) and ‘AI’ clusters (columns), measured by the ARI and AMI metrics (first and second numbers in each cell, respectively). AI clusters are generated by applying spherical k-means to text embedding vectors produced by the OpenAI-large model. Expert clusters are defined by the PRAiS2 risk model. See Data Preparation for further details.

Expert clusters	AI clusters	
	Diagnosis	Operation
Diagnosis (BDG)	**0.31 / 0.44**	0.21 / 0.32
Operation (TSP)	0.27 / 0.40	**0.29 / 0.48**

Further evidence for sensibility of AI clusters is that AI and expert clusters corresponding to the same text field (diagonal elements of Table 2, highlighted in **bold**) are more consistent than clusters based on non-matching fields. At the same time, the fact that non-matching clusters also have non-zero consistency can be explained by the correlation between a patient’s diagnoses and the operations performed on them.

**Figure 3:**
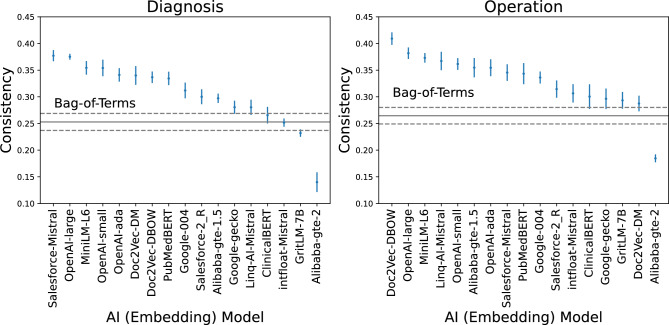
Consistency of AI and expert clusters based on diagnosis (left) and operation (right) text fields. Consistency is defined as the average of ARI and AMI scores between AI and expert data partitions. Confidence intervals are based on the 10 repeats of k-means clustering, each using 10 random initialisations of centroids. For Bag-of-Terms (BoT), we used standard k-means, while spherical k-means was used for AI embeddings. Horizontal lines represent mean (solid line) and 95% confidence intervals (dashed lines) for the BoT method. Error bars for each embedding model also represent the 95% CI.

Figure 3 shows the average ARI and AMI scores for diagnosis and operation fields using embeddings produced by different LLMs. The mean and 95% confidence intervals for the BoT approach are included as horizontal lines for comparison. We see that the majority of LLMs produce clusters that have higher consistency with expert clusters, compared with BoT. OpenAI-large has the highest combined ranking across both text columns, i.e., most consistency with experts. Since BoT - unlike domain experts—does not use semantic information contained in the description of each medical code, is not context aware and does not use the information captured in the order of entries of multiple codes for a single patient, our results suggest that LLMs are successful at extracting medically-relevant information using the semantics and the context provided by the entire text string, including the order of code entries. This is consistent with previous research^26,27^.

We asked OpenAI’s gpt-4-turbo model to summarise and explain the differences between AI and expert clusters. As seen in the excerpt below, it produced an insightful and plausible answer:

**Table:** 

*Partition 2 [experts] appears more segmented, potentially dividing patients by broader surgical categories, like major surgery types (Aortic arch repairs, Tetralogy of Fallot repairs, Norwood procedures, etc.). This suggests a more high-level clustering compared to the specific procedural focus in Partition 1 [AI] ...*	

Please note that the partition labels in square brackets are added by us; in other words, this was a blind experiment and we did not relay any information to the LLM about which data partition corresponds to AI and which one corresponds to experts. Supplementary Material A contains the full text of the prompt provided to the LLM and its response.

**Key Takeaway:** Modern LLMs are capable of extracting medically-relevant information from EHR text that is consistent with domain experts.

### Predictive performance of embeddings

**Figure 4:**
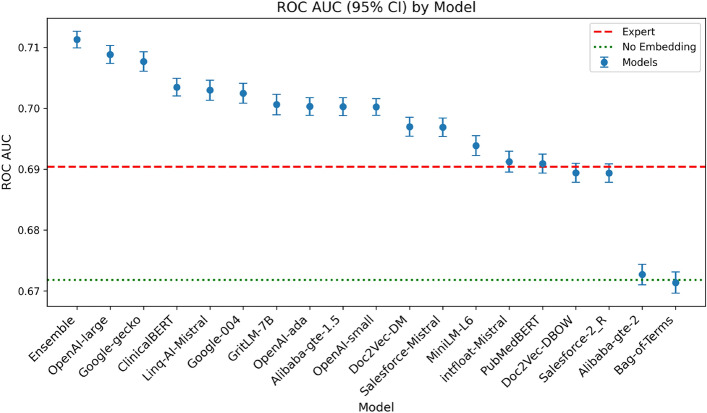
Out-of-sample ROC-AUC of binary classifiers using clusters generated—via spherical k-means—from text embedding vectors produced by various LLMs. The horizontal lines represents the performance of a baseline model that does not use any embeddings, as well as an expert-based model, which takes the average predicted probability of two binary classifiers, each using expert clusters for diagnosis and operation as categorical variables, alongside baseline attributes. ‘Ensemble Model’ predictions are a weighted average of ‘OpenAI-large’, ‘Google-gecko’ and ‘Expert’ models. Mean values and error bars are based on 250-times repeated, 10-fold cross-validation, using identical folds across all models.

Figure 4 compares the ROC-AUC for predicting ‘KDIGO-Binary’ using various embedding approaches (as well as the ‘No Embedding’ and ‘Ensemble’ options) against the ‘Expert’ model (horizontal line). Among the individual algorithms tested, OpenAI-large (second bar from left) outperforms all others, including the expert model. The ClinicalBERT model, despite not being fine-tuned for sentence embedding, shows competitive performance, being the top open-source model and only behind OpenAI and Google.

Forming an ensemble of AI and expert models further improves performance (leftmost bar). This is expected since, while AI models exhibit high correlation of fold-level errors amongst themselves, their correlation with the expert model is significantly smaller. For instance, average error correlation between pairs of AI models in the group is 92.8%, while their average correlation with the expert model is 83.0%. This is in line with the results presented earlier in Table 2 showing an imperfect consistency between AI and expert clusters.

**Table 3:** Out-of-sample performance of binary classifiers using clusters generated—via spherical k-means—from text embedding vectors produced by various LLMs. The ‘Expert’ model takes the average predicted probability of two binary classifiers, each using expert clusters for diagnosis and operation as categorical variables, alongside baseline attributes. ‘Ensemble Model’ predictions are a weighted average of ‘OpenAI-large’, ‘Google-gecko’ and ‘Expert’ models. The ‘No Embedding’ model uses only the baseline variables. Mean values and error bars are based on 250-times repeated, 10-fold cross-validation, using identical folds across all models. The ‘ROC-AUC’ column corresponds to Fig. 4. For all remaining columns, the numbers represent average of values calculated for a vector of thresholds applied to class probabilities from 0.1 to 0.9 with increments of 0.1.

Model	ROC-AUC	Accuracy	Precision	F1	Sensitivity	Specificity
Ensemble	0.711	0.680	0.419	0.251	0.320	0.804
OpenAI-large	0.709	0.681	0.417	0.254	0.322	0.805
Google-gecko	0.708	0.681	0.427	0.254	0.321	0.805
ClinicalBERT	0.703	0.678	0.388	0.243	0.315	0.803
Linq-AI-Mistral	0.703	0.678	0.391	0.243	0.316	0.803
Google-004	0.702	0.680	0.412	0.249	0.318	0.805
GritLM-7B	0.701	0.677	0.384	0.239	0.312	0.802
OpenAI-ada	0.700	0.678	0.412	0.245	0.315	0.804
Alibaba-gte-1.5	0.700	0.679	0.419	0.251	0.319	0.803
OpenAI-small	0.700	0.677	0.400	0.240	0.312	0.803
Doc2Vec-DM	0.697	0.672	0.360	0.230	0.307	0.798
Salesforce-Mistral	0.697	0.677	0.391	0.243	0.314	0.802
MiniLM-L6	0.694	0.674	0.371	0.234	0.308	0.801
intfloat-Mistral	0.691	0.675	0.370	0.233	0.309	0.801
PubMedBERT	0.691	0.674	0.389	0.235	0.307	0.800
Expert	0.690	0.673	0.391	0.235	0.308	0.799
Doc2Vec-DBOW	0.689	0.670	0.356	0.223	0.299	0.798
Salesforce-2_R	0.689	0.674	0.370	0.234	0.307	0.800
Alibaba-gte-2	0.673	0.668	0.368	0.222	0.297	0.796
No Embedding	0.672	0.663	0.330	0.207	0.288	0.793
Bag-of-Terms	0.671	0.666	0.355	0.215	0.293	0.794

Compared to the ‘No Embedding’ model, we achieved 23% improvement in discriminative power, corresponding to AUC improvement from 67.2% (baseline model) to 71.1% (the final model). As Table 3 shows, the best performing models in terms of AUC also perform well along other metrics (accuracy, precision, F1, sensitivity, and specificity). Note that, as described in Methods, all threshold-sensitive metrics are averages over a range of thresholds, from 0.1 to 0.9 with increments of 0.1. The ‘optimal’ choice of threshold is a function of the requirements of a specific application, such as the relative cost of incurring false positives and false negatives.

**Table 4:** Impact of fine-tuning on performance of models trained on clusters formed—via spherical k-means—from three open-source embedding models. P-values are based on paired t-test of fold-level errors, formed from 250-times repeated, 10-fold cross-validation. For all metrics except ROC-AUC, the numbers represent average of values calculated for a vector of thresholds applied to class probabilities from 0.1 to 0.9 ﻿with increments of 0.1. Numbers in parentheses represent the p-value—where significant—of a one-sided t-test assessing improvement in ROC-AUC after fine-tuning.

Model	Metric	Before	After	Diff
ClinicalBERT	Specificity	0.803	0.803	− 0.000
	Sensitivity	0.315	0.314	− 0.000
	ROC-AUC (0.05)	0.703	0.704	0.001
	Precision	0.388	0.394	0.006
	F1	0.243	0.243	− 0.000
	Accuracy	0.678	0.678	− 0.000
PubMedBERT	Specificity	0.800	0.802	0.001
	Sensitivity	0.307	0.309	0.002
	ROC-AUC (<1e-3)	0.691	0.694	0.003
	Precision	0.389	0.393	0.004
	F1	0.235	0.238	0.003
	Accuracy	0.674	0.675	0.002
MiniLM-L6	Specificity	0.801	0.800	− 0.000
	Sensitivity	0.308	0.308	− 0.000
	ROC-AUC	0.694	0.686	− 0.008
	Precision	0.371	0.380	0.010
	F1	0.234	0.234	0.000
	Accuracy	0.674	0.674	− 0.000

We also tested the impact of fine-tuning three open-source LLMs on their predictive performance. Results are shown in Table 4. For two of the three models tested (ClinicalBERT and PubMedBERT), there is statistically significant improvement in ROC-AUC, while for the third model (MiniLM-L6) we see performance degradation. Only in the case of PubMedBERT we see consistent improvement across all metrics after fine tuning.

**Key Takeaways:** 1) Features extracted from EHR text by most embedding LLMs—open-source and commercial—outperform features defined by domain experts when used in models for predicting AKI in paediatric CPB; 2) Fine-tuning of LLMs on EHR text can further improve the predictive performance of embeddings, although more research is needed.

### AI explainability

Average ARI/AMI scores across all 45 pairs of 10 clustering runs described in Benchmarking are 83% and 89%, respectively, indicating high stability of AI-generated clusters across runs. Similarly, after mapping the clustering runs using the algorithm described in Supplementary Material C, the average member overlap across all pairs is 86%.

Cluster labels produced by OpenAI and Google—as well as their odds-ratios—were quite consistent. Table 5 shows cluster labels for two OpenAI and two Google runs. The full mapping table is provided as a supplementary file (see Supplementary Material D).

**Figure 5:**
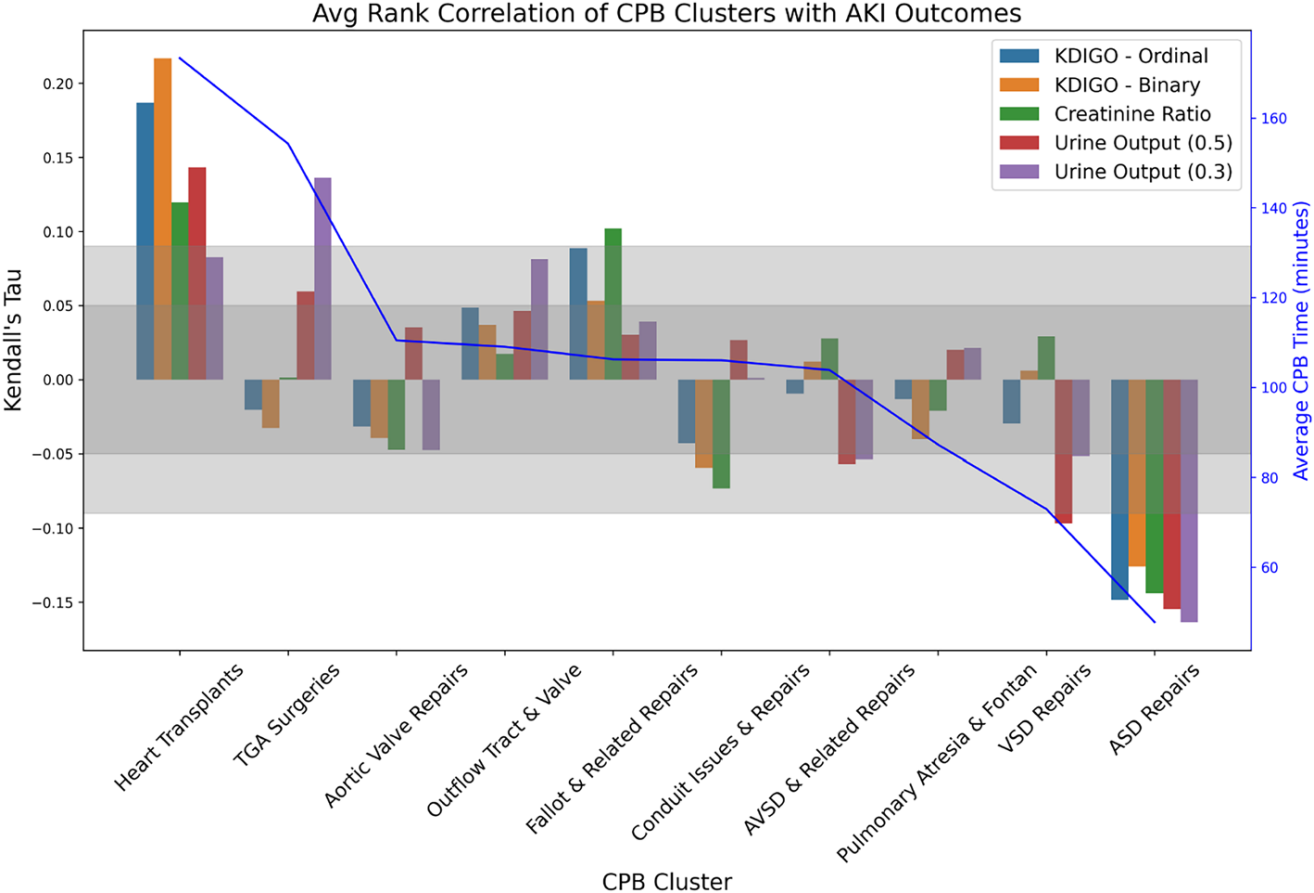
Average rank correlation (measured via Kendall’s tau) of AKI-related outcomes with membership in each of the 10 clusters defined by applying spherical k-means to a concatenation of OpenAI-large model’s embedding of diagnosis and operation text columns. Each clustering run is subsequently labelled 10 times by text-completion LLMs, five times using OpenAI’s gpt4-turbo model and five times using Google’s gemini-1.5-pro model. See “Data preparation” for definition of AKI metrics. Clusters are sorted from left to right in decreasing average operation time, which is displayed as a blue line (right y axis). Inner grey band reflects the 95% confidence interval, outside of which correlations are statistically significant. The outer grey band is similar, but after Bonferroni correction for multiple testing^36^, using 50 tests (5 outcome variables $\times$ 10 clusters). Note that cluster (x axis) labels are abbreviated versions of the longer, more descriptive cluster labels produced by the LLMs, as listed in Table 5.

**Table 5:** Comparison of two cluster labels produced by OpenAI and two by Google, each produced for clusters that were output of spherical k-means with a different set of 100 random initialisations. Cluster numbers across runs 1–3 were mapped to run 0 to maximise their member overlap using a greedy algorithm described in the Appendix. The group highlighted in Bold shows statistically significant association with increased AKI risk, while the group highlighted in Italic shows significant association with reduced AKI risk. ‘Short Label’ column matches x axis values in Fig. 4.

Short Label	OpenAI 1	Google 1	OpenAI 2	Google 2
Conduit Issues and Repairs	Cardiac Conduit Issues and Associated Repairs	Valve replacement or repair	Cardiac Conduit Failure and Replacement	Valve Replacement & Repair
Pulmonary Atresia & Fontan	Complex Pulmonary Atresia and Fontan Procedures	Univentricular heart or HLHS procedures	Complex Congenital Heart Disease	Single Ventricle Physiology
*ASD Repairs*	*Atrial Septal Defect (ASD) Repairs*	*ASD closure*	*Atrial Septal Defect (ASD) Management*	*Atrial Septal Defects*
VSD Repairs	Ventricular Septal Defect (VSD) Repairs	VSD closure	Ventricular Septal Defect (VSD) Treatment	Ventricular Septal Defects
Outflow Tract & Valve	Complex Outflow Tract Obstructions and Valve Replacements	Complex congenital heart defect repair	Anomalous Pulmonary Venous Connections	Complex Congenital Heart Defects
TGA Surgeries	Transposition of Great Arteries and Associated Surgeries	Arterial switch for TGA	Transposition of the Great Arteries (TGA) Management	Transposition of the Great Arteries
Aortic Valve Repairs	Aortic Valve Stenosis and Regurgitation Repairs	Aortic valve or arch repair	Aortic Valve and Outflow Pathway Surgery	Aortic Valve Abnormalities
AVSD & Related Repairs	Atrioventricular Septal Defect (AVSD) and Related Repairs	AVSD repair	Atrioventricular Septal Defect (AVSD)	Atrioventricular Septal Defects
**Heart Transplants**	**Heart Transplant and Advanced Cardiac Therapies**	**Transplant, assist device, or complications**	**Heart Transplant and Complications**	**Heart & Lung Transplant / VAD**
Fallot & Related Repairs	Tetralogy of Fallot and Related Repairs	Tetralogy of Fallot repair	Tetralogy of Fallot and Associated Repairs	Tetralogy of Fallot

We can gain further insight by considering how various AKI outcomes - described in Data Preparation—correlate with the AI-generated clusters. Figure 5 summarises this analysis. We make a few observations:

- Two groups show consistent, statistically significant deviation from the rest: **‘Heart Transplants’** show higher risk for all AKI outcomes vs. other CPB clusters, and *‘ASD Repairs’* show lower risk than the rest.
- For ‘TGA Surgeries’, there is a higher risk of low urine output (especially for length of time below 0.3 ml/kg/hr) that is statistically-significant, even after adjusting for multiple testing. However, this is not reflected in KDIGO outcomes and their changes are statistically insignificant.
- For ‘Fallot & Related Repairs’, serum creatinine ratio is significantly higher than the rest, and ordinal KDIGO score is also nearly significant (after multiple-testing adjustment).
- For ‘Conduit Issues & Repairs’, we see protection in terms of creatinine ratio, which is borderline significant.
- For ‘VSD Repairs’, we see significant protection in terms of urine output.

Figure 5 also plots the average cardiopulmonary bypass (CPB) time for each cluster (blue line, right y-axis), which are sorted by this metric. We observe that the cluster with the highest correlation to AKI outcomes (‘Heart Transplants’) has the longest mean CPB time, whereas the cluster with the lowest correlation (‘ASD Repairs’) exhibits the shortest mean CPB time. This relationship between CPB time and AKI risk is consistent with clinical expert intuition (co-authors of this paper) and aligns with prior meta-analytic evidence^28^.

Interestingly, the ‘Fallot and Related Repairs’ cluster demonstrates a strong correlation with elevated creatinine ratios that appears disproportionate vs. its average CPB time. This is consistent with multiple investigations focused on potential predictors of AKI in Tetralogy of Fallot surgeries^29,30^. Yet comparative studies quantifying AKI incidence in Fallot versus other CPB procedures remain limited. Specifically, the pathways distinguishing creatinine elevation from reduced urine output—both of which factor into AKI—are not well characterized and warrant further exploration.

**Key Takeaways:** 1) Modern, general-purpose LLMs can produce medically plausible summaries of clusters generated from LLM embeddings of EHR text (‘AI explaining AI’), 2) Statistical analysis of these clusters can validate/question current theories and generate hypotheses for further research.

## Discussion

This study demonstrates the value of LLMs in predicting postoperative AKI in paediatric CPB patients. The superior performance of general-purpose LLMs in such a highly specialised domain underscores their versatility and power. Prediction can be improved by forming ensembles of multiple LLMs and clinical expert models. Text-generating LLMs can successfully summarise the output of embedding LLMs, resulting in explainable AI classifications. This is a promising way to enhance trust in the use of AI prediction in medical settings and to provide deeper insights into the clinical questions^31–33^.

### Performance metrics and clinical interpretation

Several factors contribute to the relatively low absolute values observed for precision, sensitivity, and F1-score reported in Tables 3 and 4. First, the occurrence of severe AKI in our dataset ( 26%) represents an inherent class imbalance, naturally limiting the absolute values achievable for sensitivity and precision across various decision thresholds. Second, as noted in the Results section, our reported metrics are averages calculated across multiple probability thresholds (from 0.1 to 0.9). Since many of these thresholds are not optimal for maximizing these metrics, the averaged values appear lower overall. Importantly, these metrics are threshold-dependent, and there exist inherent trade-offs among sensitivity (recall), specificity, precision, and thus the F1-score. Achieving high sensitivity typically reduces precision, and vice versa, due to their reciprocal relationship influenced by threshold selection.

Additionally, our primary research objective was not maximizing absolute predictive performance but rather quantifying the relative improvement provided by incorporating unstructured textual data through embedding-based clusters. Our analyses demonstrated consistent and statistically significant improvements in model performance (particularly AUC), validating our core hypothesis about the added value of embedding features. In clinical practice, optimal thresholds would be selected based on the clinical context and the specific costs associated with false positives versus false negatives, potentially leading to considerably improved precision, sensitivity, or F1-score at operationally relevant thresholds.

### Advances in GenAI technology

Latest advances by two leading commercial providers of AI—OpenAI and Google—greatly facilitated our ‘explainability’ framework. Firstly, the increased context length of the LLMs (128k tokens for OpenAI’s gpt-4o and > 2 million tokens for Google’s gemini-1.5-pro-001) was key to our ability to execute the cluster labeling task in a single pass. This is because our prompt had in excess of 50k tokens, due to a large payload that included the text columns of interest for the entire training data. For LLMs with short context length, a potential solution would be to implement a multi-stage approach, e.g., by first creating labels for each cluster in isolation, and wrapped in a for loop, and then doing a second round of interaction with the LLM to tune the labels and make them more differentiated.

Secondly, we took advantage of the structured/JSON output modes offered by OpenAI and Google models. Enforcement of a data structure allows for automation of downstream tasks, which in our case amounted to creating summary tables showing rank correlation of membership on each cluster with outcomes. Such table summaries were the foundation for creation of Fig. 4.

### Comparison with related research

Our approach distinguishes itself from previous research on the clinical applications of LLMs in several key aspects, including benchmarking against domain experts, integrating structured features alongside EHR text, and prioritising explainability. These elements collectively enhance the interpretability and reliability of our model, ultimately facilitating broader adoption by clinical practitioners.

**Benchmarking:** The availability of expert-defined clusters (BDG and TSP) for diagnosis and operation fields enabled a rigorous benchmarking of AI-generated clusters through two key approaches: (1) computing cluster consistency metrics to assess alignment between AI and expert-derived groupings, and (2) evaluating the predictive performance of models utilising AI-generated versus expert-defined clusters. Furthermore, the standardised nature of medical terminology allowed us to employ BoT embeddings as an additional benchmark for LLM-generated embeddings. While studies such as^10^ leveraged free-text medical notes, which may capture a broader spectrum of clinical nuances, our study’s benchmarking framework provides greater interpretability and facilitates trust in AI-driven predictions.

**Structured Features:** Our inclusion of structured variables, such as age, BMI, and operation duration, enhances the real-world applicability of our results. These structured features are typically available in most clinical settings, making our predictive models more relevant for real-world applications. In contrast, studies such as^10^ focused solely on embeddings derived from textual data, which, while powerful, may limit the integration of models into existing clinical workflows that rely on structured inputs.

**Explainability:** Our focus on maximising the interpretability of text embeddings has motivated—at least in part - technical decisions such as (1) applying clustering to embeddings and using cluster memberships as low-dimensional features in the predictive model, and (2) devising a method for reverse-mapping of the embedding space to the natural-language domain. To our knowledge, this is a novel contribution.

### Applications in healthcare

Our decision to apply clustering to embedding vectors was motivated not only by mathematical properties, but also practical considerations in terms of explainability and predictive performance, both being important in healthcare settings. The cluster labels replace nameless, high-dimensional embedding vectors with interpretable features, enhancing explainability. On the other hand, by using soft membership of the same clusters, we further improve the predictive performance while still maintaining the link between the predictors and the explainable output. Active involvement of two paediatric cardiac surgeons among our co-authors - who helped verify the plausibility of the discovered clusters - adds to our confidence that the method captures clinical nuances in a manner consistent with medical expertise.

Besides explainability and predictive accuracy, scalability and generalisability are also important for implementing deicsion support systems across diverse healthcare settings^34^. Our experiments focused on paediatric cardiopulmonary bypass (CPB) and leveraged a combination of structured features and LLM-derived embeddings of unstructured text. The inclusion of structured variables (e.g., demographic and operative details) in our predictive pipeline allows it to be applicable to a wide range of clinical specialties.

Other clinical contexts may present lengthier free-text inputs (e.g. extensive clinical notes). In such scenarios, LLMs with sufficiently large context windows or multi-stage strategies can handle bigger volumes of text without degrading predictive accuracy. Although employing LLMs with extensive memory capacity has, until recently, been a technical hurdle, the rapid evolution of large-scale AI architectures suggests this barrier will continue to diminish.

Furthermore, by making available our software that encapsulates our workflow (from text embedding to clustering and explainability), we will endeavour to widen the adoption of our approach by clinicians, data scientists, and hospital IT departments. This next step would help ensure that the lessons learned in paediatric CPB—especially regarding text-based feature engineering, semantically meaningful clusters, and interpretability—can be transferred to other domains where timely, accurate predictions of postoperative or other clinical complications are equally critical.

### Future work

The promising results of this research pave the way for several future directions.

While the framework has been reviewed and validated by the surgeon co-authors, a systematic evaluation of its clinical acceptability through a qualitative study is warranted to further assess its practicality and adoption in real-world settings.

Our experiments with fine-tuning demonstrate its potential in enhancing predictive performance. The chosen fine-tuning task—associating diagnoses with corresponding operations—provides a reasonable starting point given that fine-tuning was not the primary focus of this study. Future research could explore alternative fine-tuning strategies, including supervised approaches that incorporate AKI outcomes to further optimise performance.

In our experiments, we utilised the full-length embedding vectors in downstream clustering algorithms. Future work could investigate the impact of dimensionality reduction on clustering performance. However, given that many recent embedding models are trained using the Matryoshka loss function^35^, a separate dimensionality-reduction step may not be necessary. Nevertheless, determining the optimal number of dimensions remains an important hyperparameter that could be fine-tuned using systematic search techniques, such as grid search. Similarly, the selection of the number of clusters could benefit from automated hyperparameter tuning methods.

Although we have demonstrated the value of Generative AI in predictive modeling, adoption of LLMs in clinical settings may be hindered by perceived or real technical barriers. To facilitate broader accessibility, we plan to release a Python implementation of key components discussed in this study, including text embedding, spherical k-means clustering, and LLM-based cluster interpretation.

## Conclusion

Rapid advances in quality, accessibility and user-friendliness of modern LLMs indicate a promising future for their applications in predictive analytics, including in specialised medical domains. The ability of LLMs to match or surpass clinical expert models, coupled with explainability, indicates the emerging role of GenAI in clinical research and practice. Future research will focus on applying our lessons and techniques in other medical domains, and exploring additional strategies to further enhance their explainability and predictive performance.

## Use of generative AI in the writing process

During the preparation of this work the authors used ChatGPT for editing the manuscript. After using this tool, the authors reviewed and edited the content as needed and take full responsibility for the content of the published article.

## Glossary

- **Acute Kidney Injury (AKI):** A sudden decrease in kidney function, often occurring after surgery, particularly in paediatric patients undergoing cardiopulmonary bypass (CPB). KDIGO defines a protocol for quantifying AKI using serum creatinine and urine output measurements.
- **Adjusted Mutual Information (AMI):** A measure of agreement between two clusterings, adjusted for chance, based on the mutual information between the clusterings.
- **Adjusted Rand Index (ARI):** A metric used to measure the similarity between two data clusterings, adjusted for the chance grouping of elements.
- **Area Under the Receiver Operating Characteristic Curve (ROC-AUC):** A performance measurement for classification models at various threshold settings, indicating the ability of the model to distinguish between classes.
- **Bag-of-Terms (BoT):** A text embedding technique where each medical term in a patient’s record is represented as a binary indicator in a vector.
- **Bidirectional Encoder Representations from Transformers (BERT)**: A model for natural language processing developed by Google that learns bi-directional representations of text^37^.
- **Broad Diagnosis Group (BDG)**: Mapping of the collection of diagnoses for a patient to a smaller set of diagnosis groups, by the PRAiS2 protocol.
- **Cardiopulmonary Bypass (CPB):** A technique used during heart surgery where a machine temporarily takes over the function of the heart and lungs, allowing surgeons to operate on a still heart.
- **Cross-Validation (CV):** A statistical method used to estimate the performance of machine learning models, where the data is split into multiple folds, and the model is trained and validated on different folds.
- **Doc2Vec:** A text embedding technique that learns distributed representations of documents, allowing for the transformation of entire documents into fixed-length vectors.
- **Ensemble Model:** A machine learning technique that combines the predictions of multiple models to improve accuracy and robustness.
- **Explainability:** Techniques used to interpret and understand the predictions made by complex machine learning models, often to increase trust and provide insights into the decision-making process.
- **Fine-Tuning:** The process of adjusting a pre-trained model on a new dataset, typically with a smaller learning rate, to adapt the model to a specific task or domain.
- **Hyperparameters:** Parameters of a machine learning model that are set before training and control the learning process, such as the number of clusters in k-means or the learning rate in neural networks.
- **KDIGO:** Kidney Disease Improving Global Outcomes; a set of guidelines used to define and classify the severity of acute kidney injury (AKI)^19^.
- **Large Language Models (LLMs):** Advanced machine learning models, often based on transformer architectures, that are trained on vast amounts of text data and can perform a variety of natural language processing tasks.
- **Massive Text Embedding Benchmark (MTEB):** A massive benchmark for measuring the performance of text embedding models on diverse embedding tasks.
- **Medical Information Mart for Intensive Care (MIMIC-III)**: A large, freely-available database comprising deidentified health-related data associated with over forty thousand patients who stayed in critical care units of the Beth Israel Deaconess Medical Center between 2001 and 2012.
- **Partial Risk Adjustment in Surgery (PRAiS2):** A model used in the UK to predict 30-day mortality risk after paediatric heart surgery, incorporating various clinical variables.
- **Spherical K-Means:** A variant of the k-means clustering algorithm that uses cosine distance instead of Euclidean distance, making it suitable for clustering high-dimensional data like text embeddings.
- **Text Embedding:** A method of converting text into numeric vectors that capture the semantic meaning of the text, used in machine learning models for various predictive tasks.
- **Transformed Specific Procedure (TSP)**: Mapping of the collection of procedures for a patient to a smaller set of procedure groups, by the PRAiS2 protocol.

## Supplementary Information

Supplementary Information 1.

Supplementary Information 2.

Supplementary Information 3.

## Data Availability

The data used and/or analysed during the current study are available from the corresponding author(s) upon reasonable request.
