# Supplementary material for: GenAI exceeds clinical experts in predicting acute kidney injury following paediatric cardiopulmonary bypass
Source: Sci Rep. 2025 Jul 1;15:20847. doi: 10.1038/s41598-025-04651-8 (PMC12216426; doi:10.1038/s41598-025-04651-8)
Supplement: Supplementary file 1 — Supplementary Information 1. [file 41598_2025_4651_MOESM1_ESM.pdf]

Supplementary Material - GenAI Exceeds Clinical Experts in Predicting Acute Kidney Injury following Paediatric Cardiopulmonary Bypass

Mansour Sharabiani [Corresponding author: mt5605@imperial.ac.uk][a], Alireza Mahani [Corresponding author: alireza.s.mahani@gmail.com][b], Alex Bottle[a], Yadav Srinivasan[c], Richard Issitt[c], Serban Stoica[d]

[a] *Department of Primary Care and Public Health, Imperial College London, UK*
[b] *Statman Solution Ltd., 128 City Road, London, EC1V 2NX, UK*
[c] *Great Ormond Street Hospital, London, UK*
[d] *Bristol Royal Hospital for Children, Bristol, UK*

A. AI Explaining AI - Part 1

A.1. Prompt

The following shows the prompt preamble and sample data that follows it, asking OpenAI's `gpt-4-turbo` model to interpret the difference between two partitions of patients according to their planned operations. One partition was generated by applying spherical k-means to embeddings of the `operation` field by the `OpenAI-large` embedding model ('AI cluster'). The other partition was created by using the PRAiS v2 protocol ('expert cluster'). As can be seen below, the prompt did not reveal to the LLM which partition corresponded to AI/expert cluster:

> A group of pediatric patients have undergone cardiopulmonary bypass surgery (CPB). For each patient, we have one or more descriptions concatenated via ';' into a single sting. Each patient is printed on a single line. The descriptions represent one or more surgical procedures performed during the CPB. The dataset has been partitioned into 15 groups by two different clustering methods (Partition 1 and Partition 2), based on the aforementioned text entries for the patients. Clusters of patients in each partitioning scheme are separated by multiple blank lines. The two partitioning of patients are printed below. Note that the input to

the two partitioning schemes has been an identical set of patients, with each patient having the same exact text entry in both cases. Please provide a brief interpretation of the most salient differences between the two partitions:

Partition 1:

Atrial septal defect (ASD) secundum closure with direct suture
Atrial septal defect (ASD) secundum closure with patch
Atrial septal defect (ASD) secundum closure with patch;Pacemaker system placement: permanent epicardial
Sinus venosus defect (ASD) closure
Atrial septal defect (ASD) secundum closure
Coronary sinus interatrial communication (ASD) repair
Atrial septal defect (ASD) secundum closure with patch;Atrial septal defect (ASD) secundum closure with direct suture
Atrial septal defect (ASD) secundum closure with patch;Pericardial window creation
Atrial septectomy
ATRIAL SEPTAL DEFECT (ASD) SECUNDUM CLOSURE WITH PATCH
Atrial septal defect (ASD) secundum closure with direct suture;Aortopexy

Subaortic obstruction relief
Left ventricular outflow tract obstruction relief;Implantable cardioverter & defibrillator (ICD) implantation: epicardial
Right ventricular outflow tract obstruction relief
...
...

==================================

Partition 2:

Supravalvar aortic stenosis repair
COR TRIATRIATUM (DIVIDED LEFT ATRIUM) REPAIR; ATRIAL SEPTAL DEFECT (ASD) SECUNDUM CLOSURE WITH PATCH
Closure of multiple ventricular septal defect (VSD)s
Supravalvar aortic stenosis repair;Pulmonary valvotomy: open

Aortic valvotomy: open;Supravalvar aortic stenosis repair
Rastelli procedure: intraventricular left ventricle to aorta tunnel & right ventricle to pulmonary artery conduit;Ventricular septal defect (VSD) closure using patch;120102. Atrial septal defect (ASD) secundum closure with direct suture
Supravalvar aortic stenosis repair;Aortic arch repair
Cor triatriatum (divided left atrium) repair
COR TRIATRIATUM (DIVIDED LEFT ATRIUM) REPAIR

Mitral valvar procedure
Ross procedure: aortic valve or root replacement with pulmonary autograft & pulmonary valvar replacement
Subaortic obstruction relief
...
...

A.2. LLM Response

Below is the full text of OpenAI's `gpt-4-turbo` response to the above prompt:

> Partition 1 and Partition 2 present two different clustering methodologies based on a range of complex cardiopulmonary bypass (CPB) surgeries. Here are some observations that highlight differences in the clustering approach and organization of these partitions:
>
> 1. **Partition Focus**: - **Partition 1** appears to group data based on specific types and complexities of surgeries, with more focus on nuanced relationships between different surgeries common in pediatric cardiology (e.g., combinations of ASD repair methods, valve repairs, and VSD closures). - **Partition 2** appears more segmented, potentially dividing patients by broader surgical categories, like major surgery types (Aortic arch repairs, Tetralogy of Fallot repairs, Norwood procedures, etc.). This suggests a more high-level clustering compared to the specific procedural focus in Partition 1.
>
> 2. **Detail of Procedure Combination**: - **Partition 1** shows detailed combinations of surgeries, indicating a clustering based

on specific multi-procedural patient cases (e.g., ASD closure combined with VSD closure and pulmonary artery reconstructions). - **Partition 2** while still detailed, often seems to place cases in broader procedural categories rather than intricate combinations, hinting at clustering by primary or most substantial procedure types.

3. **Repetition and Organization**: - **Partition 1** presents a form of clustering where similar procedures are often grouped together irrespective of the additional minor procedures, likely showing clusters based on primary intervention. - **Partition 2** regularly reuses certain procedures across different clusters, possibly categorizing based on a combination of the surgical procedure and some other clinical factors like patient demographics or surgical outcomes.

4. **Consistency and Case Sensitivity**: - Both partitions notably mix cases with varying capitalizations and details in the procedure descriptions, which suggests that the clustering isn't strictly dependent on textual exactness but rather on the procedural content. However, **Partition 1** seems to tolerate more variations within clusters, while **Partition 2** may be using a stricter or different set of criteria likely incorporating additional clinical variables beyond just procedural similarity.

5. **Cluster Titles and Patient Outcomes**: - Neither partition explicitly titles their clusters, which requires interpretation based on the listed procedures alone. However, the clustering logic in **Partition 2** may hint at broader outcome-based or pathology-based groupings (such as focusing on all patients undergoing arterial switch operations, regardless of accompanying procedures).

6. **Granularity**: - **Partition 1** tends to have finer granularity, focusing on the specific procedural nuances. **Partition 2**, while detailed, appears geared towards broader surgical categories, potentially suited for analyses like resource allocation or broad outcome studies.

The differences suggest diverse utility: **Partition 1** is more suitable for studies needing detailed procedural interconnections

(useful in operational research, detailed post-operative complications studies) while **Partition 2** could better serve broad-brush analyses such as policy-making, general clinical outcomes, or resource planning. The choice between partitions would depend on the specific needs of the research or administrative tasks at hand.

B. LLM Structured Output

In this section, we present the details of how the structured output features of OpenAI and Google LLM were used when requesting cluster labels, based on the descriptions of diagnosis and operation codes associated with each CPB.

B.1. OpenAI

OpenAI's structured output feature is an evolution of their JSON mode. As of this writing, it is available for `gpt-4o-mini-2024-07-18` or later, or for `gpt-4o-2024-08-06` or later. See here for details.

To use this feature, we define the classes that capture what we expect to get back for each cluster. Here, we indicate to the LLM that we want to get 1) the group number (consistent with the group numbers provided in the prompt body), 2) short description of each group, 3) long description of each group. Optionally, one can add specific character/word limits for the short and long descriptions in the prompt instructions (see B.3), or otherwise modify the template as necessary for each project:

```
class GroupLabel(BaseModel):
    group_number: int
    description_short: str
    description_long: str

class MultipleGroupLabels(BaseModel):
    groups: list[GroupLabel]
```

We can now submit a completion request to OpenAI by passing in the class `MultipleGroupLabels`:

```
response = openai_client.beta.chat.completions.parse(
    model = 'gpt-4o'
    , messages = [
        {"role": "system", "content": prompt_instructions},
        {"role": "user", "content": prompt_body}
    ]
    , response_format=MultipleGroupLabels
)
```

The arguments `prompt_instructions` and `prompt_body` are discussed further down.

B.2. Google

For Google, we use a JSON template to convey our expectations about the response structure. Below is the schema definition:

```
response_schema = {
    "type": "object",
    "properties": {
        "groups": {
            "type": "array",
            "items": {
                "type": "object",
                "properties": {
                    "group_number": {"type": "integer"},
                    "description_short": {"type": "string"},
                    "description_long": {"type": "string"},
                },
                "required": [
                    "group_number", "description_short"
                    , "description_long"
                ],
            },
        },
    },
    "required": ["groups"],
}
```

which is included in the following request:

```
response = model.generate_content(
    prompt_full
    , generation_config = GenerationConfig(
        response_mime_type = "application/json"
        , response_schema = response_schema
    )
)
```

In the above, `model` is a properly instantiated `GenerativeModel` model defined in the `vertexai.generative_models` module, and `prompt_full` is the concatenation of `prompt_instructions` and `prompt_body`, discussed next.

B.3. Prompt

The prompt consists of two sections: 1) the preamble or instructions to the LLM, and 2) the body, containing data in numbered clusters that must be described by the LLM. Below is the instructions paragraph, corresponding to the case where clusters have been created using both `diagnosis` and `operation` fields:

Prompt Instructions

The following is a list of 963 pediatric patients undergoing cardiopulmonary bypass. Each row contains one or more surgical procedures, separated by ';'. These are followed by one or more diagnoses, also separated by ';'. Patients have been grouped into 10 groups, according to their diagnoses and procedures. Please suggest group labels that are representative of their members, and also distinct from each other:

The body consists of group (or cluster) number, followed by observations in that group. For each observation, the value of the relevant text field(s) is are printed. In cases where we combine multiple text fields, we preface each text field with the name ofthe field. Below is an excerpt to illustrate the point. (*Italics* and **boldface** are added for highlighting in the manuscript, and prompts submitted to LLMs are plain text.)

Prompt Body

Group 1:

Operations: aortic root replacement: valve sparing technique || *Diagnoses*: aortic regurgitation; congenital anomaly of aortic valve; doubly committed juxta-arterial ventricular septal defect (vsd) with anteriorly malaligned fibrous outlet septum and perimembranous extension
Operations: scimitar syndrome (partially anomalous pulmonary venous connection) repair || *Diagnoses*: partial anomalous pulmonary venous connection of scimitar type
Operations: aortic valvar replacement using mechanical prosthesis; left ventricular outflow tract obstruction relief || *Diagnoses*: aortic regurgitation; lv outflow tract obstruction; aortic valvar stenosis - congenital; discordant va connections (tga); superior caval vein (svc) abnormality
Operations: mitral valvar replacement; mitral valvar annuloplasty || *Diagnoses*: mitral regurgitation
Operations: mitral valvar procedure || *Diagnoses*: mitral regurgitation; mitral valvar abnormality; mitral valvar prolapse
...
...

=======

Group 2:

Operations: vsd closure; patent arterial duct (pda) closure: surgical || *Diagnoses*: perimembranous central ventricular septal defect (vsd); patent arterial duct (pda)
...
...

C. Cluster Mapping Algorithm

```
def map_clusters(cluster1_labels, cluster2_labels, N):
    # Calculate the size of each cluster in cluster1
    cluster1_sizes = [(i, np.sum(cluster1_labels == i)) for i in range(N)]
    # Sort clusters by size in descending order
    cluster1_sizes.sort(key=lambda x: x[1], reverse=True)
    sorted_cluster1_indices = [i for i, size in cluster1_sizes]

    mapping = [-1] * N  # Initialize the mapping array with -1
    remaining_clusters2 = set(range(N))

    total_overlap = 0

    for i in sorted_cluster1_indices:
        max_overlap = -1
        best_pair = -1

        for j in remaining_clusters2:
            # Count common elements between cluster1[i] and cluster2[j]
            overlap = np.sum((cluster1_labels == i) & (cluster2_labels == j))

            if overlap > max_overlap:
                max_overlap = overlap
                best_pair = j

        # Map cluster i in cluster1 to best_pair in cluster2
        mapping[i] = best_pair
        remaining_clusters2.remove(best_pair)
        total_overlap += max_overlap

    return np.array(mapping), total_overlap
```

Figure C.1: Python implementation of an algorithm - created by the authors - for mapping two data partitions to maximise total member overlap. The algorithm is 'greedy' since it proceeds down a sorted list of clusters in one partition to achieve an $O(N^2)$ scaling behaviour, instead of $O(N!)$, where N is the number of clusters in each partition.

Figure C.1 lists the Python implementation of our algorithm for mapping clusters between two different clustering results. It uses a greedy, $O(N^2)$, approach for maximising the overlap between corresponding clusters in the two sets. An exhaustive search of all possible mappings between two clusters would be an $O(N!)$ solution. Instead, our algorithm sorts one cluster set in

descending size, and begins matching the second cluster set to the first one by one, in each step searching among the remaining clusters of the second set to find one that has maximum overlap with the cluster of focus in the first set.

This algorithm is particularly useful in evaluating the stability of clustering algorithms or comparing the results of different clustering methods. By mapping clusters based on their overlap, it provides a quantitative way to assess how similar two clustering results are, which is essential in fields like data mining, bioinformatics, and pattern recognition.

D. Cluster Mapping Full Results

The table containing the full set of results for mapping of cluster labels across ten clustering runs has been provided as *cluster_mapping_full_results.csv*. It is similar to Table 5 of the main manuscript, but it has 10 columns of cluster labels, five produced by OpenAI's `gpt-4o` model (columns ending in even digits) and five produced by Google's `gemini-1.5-pro` model (columns ending in odd digits).

E. Full Names and Revision of Hugging Face Models

The below table provides the full names and revision numbers for the Hugging Face models used in the study. Note that 'ClinicalBERT' is not an embedding model out of the box. To use this model for embedding tasks, we applied mean pooling to the last hidden state of the model, which represented token-level embeddings, to arrive at sentence/document-level embeddings.

Short Name	Full Name (on Hugging Face)	Revision (Commit Hash)
Salesforce-Mistral	Salesforce/SFR-Embedding-Mistral	938c560d1c236aa563b2dbdf084f28ab28bccb11
MiniLM-L6	sentence-transformers/all-MiniLM-L6-v2	8b3219a92973c328a8e22fadcfa821b5dc75636a
PubMedBERT	NeuML/pubmedbert-base-embeddings-matryoshka	58bbcf26677463d97005dbcf45674251c3b6460e
SFR-Embedding-2_R	Salesforce/SFR-Embedding-2_R	91762139d94ed4371a9fa31db5551272e0b83818
Alibaba-gte-1.5	Alibaba-NLP/gte-Qwen1.5-7B-instruct	53a17ec94db2682f967c0ab228c55a056bf767ed
Linq-AI-Mistral	Linq-AI-Research/Linq-Embed-Mistral	0c1a0b0589177079acc552433cad51d7c9132379
ClinicalBERT	medicalai/ClinicalBERT	3bb5faa9f33458dd7801549e88767c3b23264942
intfloat-Mistral	intfloat/e5-mistral-7b-instruct	07163b72af1488142a360786df853f237b1a3ca1
GritLM-7B	GritLM/GritLM-7B	13f00a0e36500c80ce12870ea513846a066004af
Alibaba-gte-2	Alibaba-NLP/gte-Qwen2-7B-instruct	a3b5d14542d49d8e202dcf1c382a692b1607cee5

Table 1: List of full names and revisions for the Hugging Face models used in this study. The first column corresponds to the handle used for each model in the figures and tables of the main text.
